# Antimicrobial Prescribing before and after the Implementation of a Carbapenem-Focused Antimicrobial Stewardship Program in a Greek Tertiary Hospital during the COVID-19 Pandemic

**DOI:** 10.3390/antibiotics12010039

**Published:** 2022-12-27

**Authors:** Nikolaos Spernovasilis, Evangelos I. Kritsotakis, Anna Mathioudaki, Alexandra Vouidaski, Ioulia Markaki, Despoina Psaroudaki, Petros Ioannou, Diamantis P. Kofteridis

**Affiliations:** 1 School of Medicine, University of Crete, 71500 Heraklion, Greece; 2 Department of Internal Medicine and Infectious Diseases, University Hospital of Heraklion, 71500 Heraklion, Greece; 3 Department of Infectious Diseases, German Oncology Center, 4108 Limassol, Cyprus; 4 Laboratory of Biostatistics, School of Medicine, University of Crete, 71500 Heraklion, Greece

**Keywords:** point prevalence survey, carbapenems, antimicrobial stewardship, COVID-19

## Abstract

Background: Irrational use of antimicrobials poses a significant risk for public health by aggravating antimicrobial resistance. The aim of this repeated point prevalence survey (PPS) was to evaluate the impact of a carbapenem-focused antimicrobial stewardship program (ASP) on overall antimicrobial use and quality of antimicrobial prescribing during the COVID-19 pandemic. Methods: All adult inpatients in the University Hospital of Heraklion in Greece were audited twice, before and after the implementation of the ASP, in October 2019 and October 2020, respectively. Patient characteristics, indications and diagnoses for antimicrobial administration, antimicrobials prescribed, and compliance with treatment guidelines were recorded. Results: Of 743 adult inpatients on the days of the two surveys, 398 (53.6%) were on antimicrobials for 437 diagnoses. Following implementation of the ASP, there was substantial decrease in the utilization of carbapenems (4.9% of all antibacterials prescribed in the second PPS compared to 10.3% in the first PPS). A significant improvement was observed for all indicators of the quality of antimicrobial prescribing. Conclusions: Our study demonstrated a positive impact of an ASP implementation during the first stages of the COVID-19 pandemic on reducing the use of last-line antimicrobials and improving overall quality of antimicrobial prescribing.

## 1. Introduction

Antimicrobial overuse and misuse represent major public health problems worldwide and are tightly linked with negative patient outcomes, emergence and spread of antimicrobial resistance (AMR), increased risk of side effects, and higher healthcare cost [1,2]. The COVID-19 pandemic aggravated the issue of inappropriate antimicrobial use in several ways. Specifically, in many cases antibiotics were used irrationally to treat COVID-19 patients without proof or suspicion of bacterial co- or superinfection, and antiparasitics were often used as repurposed drugs against SARS-CoV-2 in the absence of scientific evidence [3,4,5].

For many years now, Greece ranks among the European countries with the highest rates of antibiotic consumption and AMR, both in community and hospital settings, and is one of the largest consumers of last-line antibiotics, such as carbapenems and polymyxins [6,7]. Implementation of targeted efforts, based on local data, is imperative for improvement of antimicrobial use. These efforts should aim to various levels of the antimicrobial prescription chain, including prescriber education, prescription practices, patient monitoring and feedback, and communication [8]. Relatively little investment per capita in infection prevention and control (IPC) strategies and antimicrobial stewardship programs (ASPs) could pay itself back in a very short time by reducing the burden of disability and death due to infections caused by multidrug-resistant bacteria [9]. Currently, a national action plan on AMR is under development, while few Greek hospitals have already attempted to optimize IPC practices and to implement ASPs [10,11,12,13].

On the 1 January 2020, a carbapenem-focused ASP was implemented in all adult clinics of our hospital in Greece. The program was based on the prospective audit and feedback strategy, along with case-based education and meetings on proper use of antimicrobials. The ASP team was alerted by the hospital pharmacy upon prescription order of a carbapenem and, within 72 h, provided unsolicited in-person consultation.

In parallel to the carbapenem-focused ASP in our hospital, repeated point prevalence surveys (PPS) were performed among all adult inpatients, aiming to identify risk factors associated with inappropriate antimicrobial use in our hospital and to evaluate the impact of the ASP on overall antimicrobial utilization and quality of antimicrobial prescribing.

## 2. Results

In all, 743 patients were hospitalized on the days of the two surveys, of whom 398 patients (53.6%) were receiving antimicrobials for 437 diagnoses. Of the 398 inpatients surveyed in the 2019 PPS, 203 (51.0%) were on antimicrobials, while in the 2020 PPS, 195 (56.5%) of the 345 inpatients were receiving antimicrobials. Baseline characteristics of patients on antimicrobials are presented in Table 1.

The majority of antimicrobial prescriptions was for therapeutic reasons. The most common indication for antimicrobial treatment was community-acquired infection (CAI) followed by hospital-acquired infection (HAI), while between the two surveys there was a statistically significant difference regarding indications for antimicrobial administration. The top three diagnoses for antimicrobial prescription in both PPSs were respiratory infections, followed by skin, soft tissue, bone and joint infections, and gastrointestinal infections (including intra-abdominal and *Clostridioides difficile* infections) (Table 2).

No statistically significant differences were observed between the two survey periods regarding the frequency of use of different antimicrobial types (Table 3). Antibacterials were the most common antimicrobials prescribed, followed by antifungals. In both PPSs, cephalosporins were the most commonly prescribed antibacterials, while fluoroquinolones and penicillins ± β-lactamase inhibitors alternated in the second and third position of the most commonly prescribed antibacterials (Table 4). Importantly, after the implementation of the carbapenem-focused ASP, there was substantial decrease in the utilization of carbapenems (4.9% of all antibacterials prescribed in the second PPS compared with 10.3% in the first PPS). Apart from McCabe score, no significant differences were observed regarding the characteristics of the patients receiving carbapenems in the two surveys (Table 5). This decrease in carbapenem use after the ASP implementation was also accompanied by a decrease in colistin use and an increase in piperacillin/tazobactam and tigecycline utilization, while the use of cephalosporins + β-lactamase inhibitors (i.e., ceftolozane/tazobactam and ceftazidime/avibactam) remained largely unchanged (Table 4).

Regarding the quality of antimicrobial prescribing, a statistically significant improvement was observed in all relative indicators after the implementation of the carbapenem-focused ASP in our hospital (Table 6). The rate of documentation in patient notes of reason and of stop/review date of antimicrobial administration was significantly higher (*p* < 0.001) in the second PPS, while full compliance to national or international treatment guidelines was also significantly increased from 61.8% to 73.6% (*p* = 0.003) after ASP implementation.

## 3. Materials and Methods

### 3.1. Study Design and Study Site

The first and second PPSs were conducted in October 2019 and October 2020, respectively. All adult wards of the University Hospital of Heraklion in Greece, a 770-bed hospital that covers all medical and surgical specialties, were audited. The study was approved by the hospital review board.

### 3.2. Study Population

All adult inpatients who were in the ward at 08:00 a.m. were audited for receipt of antimicrobials, including antibacterials, antifungals, antivirals, and antiparasitics. The routes of antimicrobial administration were parenteral (i.e., intravenous, subcutaneous, intramuscular, intraventricular, and intraperitoneal), inhalation, oral, and rectal. Outpatients, patients in the emergency department, and day hospitalizations were excluded. The number of eligible patients on the day of each survey determined the study size and no a priori calculation of sample size was performed.

### 3.3. Data Collection

Each survey was conducted on a single day by the infection control team, which constituted by infectious disease fellows and internal medicine residents. Both surveys were conducted by the same infection control team members. The Global Point Prevalence Survey (Global PPS) 2019 methodology was used with adaptations for data collection on ward and patient level [14]. The required patient data were collected by reviewing patients’ case notes and prescribing charts.

Wards were grouped by type as follows: medicine, surgery, and intensive care unit (ICU). Antimicrobial utilization data is presented in terms of proportions. Numerator data included patients on at least one antimicrobial, while denominator data involved all hospitalized patients included in the surveys. For each patient receiving antimicrobials, information was collected about sex, age, body mass index (BMI), McCabe score, presence of invasive devices, therapeutic indication, diagnosis, microbiological data, antimicrobial agents, route of administration, dosage, and a set of quality indicators: documentation of reason for antimicrobial administration in notes and of stop/review date, and compliance with national or international treatment guidelines. Each patient could have more than one diagnoses for antimicrobial treatment. Treatment guidelines compliance per diagnosis was considered as partial if the choice of antimicrobial agent(s) was following existing guidelines but dosage, route of administration or duration of treatment were inappropriate, while compliance was considered as full if all of the aforementioned treatment parameters were according to relative national or international guidelines. Of note, treatment guidelines focus on diagnosis and a patient might have more than one infection diagnosed, while each infection might be treated with more than one antimicrobial. Therefore, and in contrast to most previous studies, we selected number of diagnoses as numerator and denominator (not antimicrobials or number of patients) for the above quality indicators.

### 3.4. Statistical Analysis

Categorical data were presented as frequencies and proportions (%) and were compared between the two independent surveys (2019 vs. 2020) by means of Pearson’s chi-square test. Continuous data were summarized as mean with standard deviation or median with interquartile range (IQR) depending on the degree of skewness in the distributions and were compared between 2019 and 2020 using the *t*-test and the Wilcoxon–Mann–Whitney U test, respectively. Statistical significance was considered at the *p* < 0.05 threshold. All analyses were performed using Stata version 17 (Stata Corp., College Station, TX, USA).

## 4. Discussion

This is the first study in the current literature examining the impact of a hospital-wide carbapenem-focused ASP that was implemented during the initial stages of the COVID-19 pandemic on antimicrobial utilization and quality of antimicrobial prescribing. The results of this study confirm the feasibility and effectiveness of a hospital ASP even under the difficult circumstances of a pandemic.

Among the main findings of our study is that the implementation of the carbapenem-focused ASP in our hospital led to a decrease of carbapenem use without increasing the utilization of newer antibiotics that can be used as alternatives to carbapenems, specifically ceftolozane/tazobactam and ceftazidime/avibactam, thus preserving their efficacy through prudent use. Furthermore, the ASP caused a statistically significant improvement in quality indicators of antimicrobial prescribing, specifically in indication/diagnosis and stop/review date documentation, and adherence to treatment guidelines.

The prevalence of antimicrobial use in both our surveys (51% and 56.5%) was higher than the overall prevalence rate reported for southern Europe (39%) and the weighted prevalence in the European Union/European Economic Area (30.5%) in the pre-COVID-19 era [15,16], and also higher than the prevalence reported in multi-center studies in Japan (33.5%) and Canada (33.5%) [17,18]. However, the rates of antimicrobial use in our study were similar to those previously reported for Greece (55.6%) in the most recent (2016–2017) European Centre for Disease Prevention and Control PPS [16]. Prevalence of antimicrobial use over 50% in hospitalized patients has also been reported in studies from countries outside Europe, such as Brazil (52.2%) and Nigeria (59.6%) [19,20], and in a multinational study in Latin America that examined only the use of antibiotics (54.6%) [21]. In a multicenter PPS conducted in 2015 in the United States, almost half of inpatients surveyed were on antimicrobials [22]. Interestingly, in the above study, there was no significant reduction in the prevalence of antimicrobial use from 2011, even though the majority (79.4%) of participating hospitals reported having an ASP following the Centers for Disease Control and Prevention recommendation made in 2014 that all hospitals in the USA have an ASP [22,23]. However, compared with the 2011 survey, some positive, though unrelated to the overall prevalence of antimicrobial use, changes were observed, such as a smaller percentage of patients on fluoroquinolones and a lower prevalence of antimicrobial use in neonatal critical care settings [22]. Accordingly, in our study, although there was no significant change in the prevalence of antimicrobial use between the two surveys, ASP implementation had a positive impact on utilization of last-line antibiotics and on prescribing quality.

During the first waves of the COVID-19 pandemic, increased and often inappropriate use of antimicrobials was observed in patients with COVID-19 [24]. The reported proportion of COVID-19 inpatients receiving antibiotics ranged between 6% and 58% and in most cases treatment was empirical [25,26,27]. In a recent multinational European PPS, 52.7% of hospitalized COVID-19 patients were receiving antibiotics and/or antifungals (range, 32.9–85.6%), pneumonia was the most common diagnosis, and treatment was mostly empirical [28]. Due to the low number of hospitalized COVID-19 patients on the day of the second survey of our study (data not shown), we did not perform separate analysis for these patients, although the majority (>50%) of them were on antibiotics and/or antifungals as empirical treatment for respiratory infections.

The most common indication of antimicrobial treatment was CAI, as has been observed in similar studies from all over the world, even during the pre-COVID-19 era [15,16,18,19,20,21,29,30]. Approximately 30% of total indications for antimicrobial treatment in both our surveys were HAIs, a proportion that is considered high compared to data reported from other countries [21]. There was a slight decrease in the rate of antimicrobial administration for HAIs in the second PPS of our study compared to the first (29.1% versus 31.3%); however, taking into account the strengthening of the infection control measures in our hospital due to the COVID-19 pandemic during the second survey, this only slight decrease cannot be considered as promising. Importantly, there was a decrease in the prevalence of surgical prophylaxis for more than one day among patients on antimicrobials in the second PPS, probably due to the implementation of the ASP.

In both surveys of this study, cephalosporins were the most common antibiotics prescribed, which has also been reported in Africa, Latin America, the United States, Middle East, India, and in other studies from Greece [20,21,22,30,31,32]. This is mostly due to the wide utilization of third generation cephalosporins, which are considered safe antibiotics that can be used as empirical treatment against many common bacteria in different infection sites, such as the abdomen, and the respiratory and urinary tract. The second most commonly prescribed group of antibacterials were penicillins ± β-lactamase inhibitors, while in other similar studies, particularly from northern/western Europe and Canada, these antibacterials were the most commonly used [15,29]. The third most common group were fluoroquinolones, with an unchanged percentage of utilization between the two surveys (12.9% of all antibacterials in 2019 versus 12.4% in 2020), which is higher compared to that reported in studies derived from several other countries worldwide [21,29,30]. Taking into account the association of fluoroquinolone use with adverse drug reactions and risk of *C. difficile* infection, this class of antibacterials should be included among the primary targets of stewardship efforts.

An important outcome of our study was the decrease of carbapenem use between the two surveys after the implementation of the carbapenem-focused ASP, without concomitant increase in the utilization of most of the other antibiotics for multidrug-resistant Gram-negative bacteria, such as colistin and, most importantly, ceftolozane/tazobactam and ceftazidime/avibactam. Before the COVID-19 pandemic and the ASP implementation in our hospital, carbapenem use represented 10.3% of all antibacterials, which, even similar to that reported in a recent multinational study from Latin America [21], was considered as high and, therefore, was set as the main target of our antimicrobial stewardship intervention. In the second survey, post-ASP implementation, the respective percentage fell to 4.9%. Considering that our study was conducted in a setting of high endemicity for resistant Gram-negative microorganisms, while there was healthcare personnel shortage for stewardship activities due to the COVID-19 pandemic, this is an encouraging finding towards the feasibility of implementing an effective hospital ASP in countries with high AMR rates and limited staff resources.

The implementation of the ASP in our hospital took into account selected problems regarding antimicrobial prescribing quality that were detected during the first PPS. These problems were related to compliance to treatment guidelines and indication/diagnosis and stop/review date documentation in patient files. Even though guidelines cannot always account for individual variations among patients, adherence is associated with favorable patient outcomes. In addition, reporting the reason for antimicrobial administration and of administration stop/review date in patient charts ensures communication of diagnosis and treatment among healthcare providers and allows for appropriate follow-up plans and interventions, such as antimicrobial de-escalation [15]. Before ASP implementation, the rates of full compliance to guidelines and documentation of the reason for treatment and stop/review date of treatment were considered low compared to most of other analogous studies, which, however, used a similar but not exactly the same approach for calculating these indicators [18,20,29,33]. In the second PPS, these rates were significantly improved, which indirectly reflects the effectiveness of our antimicrobial stewardship intervention. Of note, the majority (98.5%) of the doctors in our hospital were in favor of continuing and further developing the ASP during the COVID-19 pandemic [34].

The current study has several strengths and limitations. Apart from the fact that it is the first of its kind, both surveys were conducted by the same members of the infection control team, which was composed by doctors of the Internal Medicine and Infectious Diseases department of our hospital, thus minimizing bias in collecting and interpreting data. In addition, each PPS was conducted in the same season of the year, out of summer holiday, when hospital stuffing is usually low, and winter, when antimicrobial use is the highest, in order to reduce potential confounders. On the other hand, the main limitation of our study is inherent to the method used for the two cross-sectional surveys, namely the interpretation of single point data. Furthermore, this is a single center study, in a hospital whose capacity was not exceeded due to the COVID-19 pandemic, therefore the results should be generalized with caution. Finally, our intention in this repeated PPS was to detect changes in antibiotic prescribing indicators following the implementation of the carbapenem-focused ASP, but should note that prevalence percentages presented in this study may be imprecise due to the small sample sizes available per year.

## 5. Conclusions

Our study demonstrated a positive impact of an ASP implementation on the utilization of last-line antimicrobials during the first stages of the COVID-19 pandemic in a healthcare setting with high AMR rates. Even under the pressure of the pandemic, the relation between stewardship efforts and improved quality of antimicrobial prescribing was confirmed. The findings of this study provide infectious disease doctors with useful insights into the design, implementation and further development of hospital ASPs.

## Figures and Tables

**Table 1:** Patients’ characteristics, 2019 vs. 2020.

	Total	2019	2020	*p*-Value
	(*n* = 398)	(*n* = 203)	(*n* = 195)	
Female	164 (41.2%)	95 (46.8%)	69 (35.4%)	0.021
Age (years)	65.5 (49.0–78.0)	66.0 (50.0–78.0)	65.0 (48.0–78.0)	0.59
BMI (kg/m^2^)	26.0 (23.0–29.0)	25.0 (23.0–29.0)	27.0 (24.0–30.0)	0.30
McCabe score				0.006
Non-fatal	233 (58.5%)	133 (65.5%)	100 (51.3%)	
Ultimately fatal	127 (31.9%)	52 (25.6%)	75 (38.5%)	
Rapidly fatal	33 (8.3%)	13 (6.4%)	20 (10.3%)	
Missing	5 (1.3%)	5 (2.5%)	0 (0.0%)	
Treatment setting				0.19
Medical	191 (48.0%)	106 (52.2%)	85 (43.6%)	
Surgical	176 (44.2%)	81 (39.9%)	95 (48.7%)	
Intensive care	31 (7.8%)	16 (7.9%)	15 (7.7%)	
Inserted invasive devices (total)	1.0 (1.0–2.0)	1.0 (1.0–2.0)	2.0 (1.0–2.0)	0.20
Indwelling urinary catheter	164 (41.2%)	81 (39.9%)	83 (42.6%)	0.59
Peripheral vascular catheter	349 (87.7%)	182 (89.7%)	167 (85.6%)	0.22
Central vascular catheter	59 (14.8%)	29 (14.3%)	30 (15.4%)	0.76
Invasive respiratory endotracheal intubation	34 (8.5%)	15 (7.4%)	19 (9.7%)	0.40
Inserted tubes and drains	50 (12.6%)	16 (7.9%)	34 (17.4%)	0.004

Data are presented as median (IQR) for continuous measures, and *n* (%) for categorical measures.

**Table 2:** Indications and diagnoses for antimicrobial use, 2019 vs. 2020.

Indicator	2019	2020	*p*-Value
Hospitalized patients	398 (100.0%)	345 (100.0%)	-
Patients on antimicrobials ^(1)^	203 (51.0%)	195 (56.5%)	0.133
Total number of diagnoses ^(2)^	217 (54.5%)	220 (63.8%)	0.102
Indication ^(3)^			0.021
CAI	83 (38.2%)	94 (42.7%)	
SP1	1 (0.5%)	0 (0.0%)	
SP2	0 (0.0%)	10 (4.5%)	
SP3	42 (19.4%)	34 (15.5%)	
HAI	68 (31.3%)	64 (29.1%)	
MP	15 (6.9%)	15 (6.8%)	
UNK	8 (3.7%)	3 (1.4%)	
Diagnosis ^(3)^			0.142
UNK	9 (4.1%)	2 (0.9%)	
CNS	6 (2.8%)	4 (1.8%)	
EYE	0 (0.0%)	2 (0.9%)	
ENT	6 (2.8%)	6 (2.7%)	
RESP	46 (21.2%)	41 (18.6%)	
CVS	9 (4.1%)	4 (1.8%)	
GI	35 (16.1%)	37 (16.8%)	
SSTBJ	36 (16.6%)	43 (19.5%)	
UTI	30 (13.8%)	27 (12.3%)	
GUOB	12 (5.5%)	17 (7.7%)	
BAC	4 (1.8%)	13 (5.9%)	
SEPSIS	9 (4.1%)	4 (1.8%)	
FN	5 (2.3%)	5 (2.3%)	
OTHER/NDS	10 (4.6%)	15 (6.8%)	
Treatment for HAI or CAI ^(4)^			0.310
Empirical	102 (67.5%)	98 (62.0%)	
Targeted	49 (32.5%)	60 (38.0%)	

Notes: ^(1)^ percentages calculated over the total number of hospitalized patients; ^(2)^ each patient could have more than one diagnosis; ^(3)^ percentages calculated over the total number of diagnoses; ^(4)^ percentages calculated over the sum of CAIs and HAIs. Abbreviations: CAI = community acquired infection, SP1 = surgical prophylaxis 1 dose, SP2 = surgical prophylaxis for 1 day, SP3 = surgical prophylaxis > 1 day, HAI = hospital acquired infection, MP = medical prophylaxis, UNK = unknown, CNS = central nervous system infection, EYE = eye infection, ENT = ear, nose, throat infection, RESP = respiratory infection, CVS = cardiovascular system infection, GI = gastrointestinal infection, SSTBJI = skin and soft tissue and bone/joint infection, UTI = urinary tract infection, GUOB = genitourinary and obstetric/gynecological infection, BAC = bacteremia or fungemia with no clear anatomic site and no shock, SEPSIS = sepsis of any origin, sepsis syndrome or septic shock with no clear anatomic site, FN = fever in neutropenic patient, NDS = no defined site.

**Table 3:** Frequencies of antimicrobials prescribed by type, 2019 vs. 2020.

Antimicrobial Type	2019 (*n* = 343)	2020 (*n* = 348)	*p*-Value
Antibacterial	310 (90.4%)	307 (88.2%)	0.358
Antimycobacterial	0 (0.0%)	3 (0.9%)	0.085
Antifungal	25 (7.3%)	30 (8.6%)	0.518
Antiviral	7 (2.0%)	8 (2.3%)	0.816
Antiparasitic	1 (0.3%)	0 (0.0%)	0.313

Data are presented as *n* (%) of total number of antimicrobials.

**Table 4:** Type of antibacterials, 2019 vs. 2020.

	Total	2019	2020	*p*-Value
	(*n* = 617)	(*n* = 310)	(*n* = 307)	
Antibacterial group				0.094
Penicillin ± β-lactamase inhibitor	89 (14.4%)	38 (12.3%)	51 (16.6%)	
Cephalosporin	130 (21.1%)	67 (21.6%)	63 (20.5%)	
Cephalosporin + β-lactamase inhibitor	11 (1.8%)	5 (1.6%)	6 (2.0%)	
Carbapenem	47 (7.6%)	32 (10.3%)	15 (4.9%)	
Aminoglycoside	9 (1.5%)	5 (1.6%)	4 (1.3%)	
Tetracycline	1 (0.2%)	1 (0.3%)	0 (0.0%)	
Macrolide	7 (1.1%)	5 (1.6%)	2 (0.7%)	
Lincosamide	11 (1.8%)	5 (1.6%)	6 (2.0%)	
Fluoroquinolone	78 (12.6%)	40 (12.9%)	38 (12.4%)	
Trimethoprim/Sulfamethoxazole	10 (1.6%)	3 (1.0%)	7 (2.3%)	
Metronidazole	49 (7.9%)	20 (6.5%)	29 (9.4%)	
Oxazolidinone	19 (3.1%)	13 (4.2%)	6 (2.0%)	
Glycopeptide	42 (6.8%)	19 (6.1%)	23 (7.5%)	
Daptomycin	37 (6.0%)	20 (6.5%)	17 (5.5%)	
Tigecycline	25 (4.1%)	7 (2.3%)	18 (5.9%)	
Colistin	29 (4.7%)	18 (5.8%)	11 (3.6%)	
Other antibacterial	23 (3.7%)	12 (3.9%)	11 (3.6%)	

Data are presented as *n* (%) of total number of antibacterials.

**Table 5:** Characteristics of patients receiving carbapenems, 2019 vs. 2020.

	Total	2019	2020	*p*-Value
	(*n* = 44)	(*n* = 31)	(*n* = 13)	
Female	17 (38.6%)	13 (41.9%)	4 (30.8%)	0.49
Age (years)	68.0 (55.5–79.0)	68.0 (53.0–79.0)	68.0 (59.0–79.0)	0.85
BMI (kg/m^2^)	25.0 (22.0–30.0)	24.0 (21.0–30.0)	27.0 (22.0–30.0)	0.40
McCabe				0.004
Non-fatal	17 (38.6%)	16 (51.6%)	1 (7.7%)	
Ultimately fatal	18 (40.9%)	11 (35.5%)	7 (53.8%)	
Rapidly fatal	7 (15.9%)	2 (6.5%)	5 (38.5%)	
Missing	2 (4.5%)	2 (6.5%)	0 (0.0%)	
Treatment unit				0.92
Medical	29 (65.9%)	21 (67.7%)	8 (61.5%)	
Surgical	9 (20.5%)	6 (19.4%)	3 (23.1%)	
Intensive care	6 (13.6%)	4 (12.9%)	2 (15.4%)	
Inserted invasive devices (total)	2.0 (1.0–2.0)	2.0 (1.0–2.0)	2.0 (2.0–2.0)	0.14
Indwelling urinary catheter	24 (54.5%)	15 (48.4%)	9 (69.2%)	0.21
Peripheral vascular catheter	35 (79.5%)	26 (83.9%)	9 (69.2%)	0.27
Central vascular catheter	12 (27.3%)	8 (25.8%)	4 (30.8%)	0.74
Invasive respiratory endotracheal intubation	5 (11.4%)	3 (9.7%)	2 (15.4%)	0.59
Inserted tubes and drains	5 (11.4%)	1 (3.2%)	4 (30.8%)	0.009

Data are presented as median (IQR) for continuous measures and *n* (%) for categorical measures.

**Table 6:** Therapy quality indicators by diagnoses, 2019 vs. 2020.

	Total	2019	2020	*p*-Value
	(*n* = 437)	(*n* = 217)	(*n* = 220)	
Reason in notes	331 (75.7%)	130 (59.9%)	201 (91.4%)	<0.001
Stop/Review Date Documented	204 (46.7%)	49 (22.6%)	155 (70.5%)	<0.001
Guidelines Compliance				0.003
No	93 (21.3%)	47 (21.7%)	46 (20.9%)	
Yes	296 (67.7%)	134 (61.8%)	162 (73.6%)	
Not assessable	9 (2.1%)	8 (3.7%)	1 (0.5%)	
No information	11 (2.5%)	9 (4.1%)	2 (0.9%)	
Partially	28 (6.4%)	19 (8.8%)	9 (4.1%)	

Data are presented as *n* (%) of the total number of diagnoses for which an antimicrobial was prescribed.

## Data Availability

The data presented in this study are available on request from the corresponding authors.
